# Coronavirus Disease Clinical and Laboratory Parameters: Dismembering the Values Reveals Outcomes

**DOI:** 10.7759/cureus.13720

**Published:** 2021-03-05

**Authors:** Tehzeeb Zehra, Shahzad K Siddique, Rahila Aamir, Adil Mahmood, Abdul Hameed Kiani, Sana T Virk

**Affiliations:** 1 Internal Medicine, Shifa Tameer-E-Millat University, Shifa College of Medicine, Islamabad, PAK; 2 Internal Medicine, Shifa International Hospital, Islamabad, PAK; 3 Internal Medicine, Air University Islamabad, Islamabad, PAK

**Keywords:** serial laboratory testing in covid -19, covid-19, trends of laboratory values in covid-19, outcome predictors in covid-19, curtail laboratory testing in covid-19 patients

## Abstract

Background

The medical community’s understanding of the novel coronavirus disease (COVID-19) was limited initially, and many laboratory investigations were performed to observe effects of the virus on the body, its complications, and outcomes. We observed that some laboratory investigations provided redundant information regarding outcomes, and, therefore, were not necessary. Therefore, the extent of laboratory investigations may need to be pared down to not only avoid issues related to repeated blood sampling but also to minimize the financial burdens in poor socioeconomic countries.

Objective

This study aimed to observe trends of clinical and laboratory values in COVID-19 patients and their relationship to outcomes, including disease severity, length of hospital stay, and mortality.

Methods

We conducted an observational cohort study of COVID-19 patients treated as inpatients at the Shifa International Hospital (SIH) in Islamabad in April 2020. Patients were included if they were nonsurgical, adult inpatients of SIH diagnosed with COVID-19 via positive polymerase chain reaction test. We monitored study participants' clinical and laboratory values (including hypoxia) on admission and throughout the study period. We used IBM SPSS Statistics for Windows, Version 23.0 (IBM Corp., Armonk, NY, USA) for data entry and analysis. Descriptive statistics were calculated for qualitative and quantitative data. We determined the effect of all variables on outcomes through chi-squared or Fisher's exact test, and p-values <0.05 with 95% confidence interval were considered statistically significant.

Results

A total of 51 patients with COVID-19 were enrolled. Most of the study participants were men older than age 50 with multiple comorbidities and resided in Khyber Pakhtunkhwa. Length of hospital stay ranged from eight to 14 days, and most patients had severe disease and survived. Factors such as patient age, gender, comorbid conditions, residence, and medication did not significantly affect outcomes. Hypotension during the height of symptoms and oxygen saturations <80% on admission was associated with prolonged hospital stays. Two complete blood count (CBC) parameters (platelet counts and mean corpuscular volume, MCV) were strongly associated with mortality and severity in our patients. Four non-CBC parameters (alanine transaminase, ALT; D-dimer; C-reactive protein, CRP; and lactate dehydrogenase, LDH) had strong statistical impact on disease severity, length of hospital stay, and mortality in our patients.

Conclusion

In a resource-limited country, laboratory testing must be chosen wisely and used appropriately. Patient age, gender, comorbid conditions, drugs, residence, and ferritin levels did not affect COVID-19 outcomes. Hemoglobin, platelet count, MCV, CRP, D-dimer, ALT, LDH, hypoxia, and hypotension were all correlated to disease outcomes. Therefore, these factors are useful laboratory examinations for COVID-19 patients, especially in poor countries.

## Introduction

The novel coronavirus disease (COVID-19) needs little introduction since December 2019, when the healthcare workers in the city of Wuhan, China, first discovered cases at the start of the COVID-19 epidemic [1]. As of November 1, 2020, nearly 46 million cases and 1.2 million deaths have been reported globally due to COVID-19, while the pandemic continues as a Public Health Emergency of International Concern, according to the World Health Organization (WHO) [2].

While various theories have been proposed regarding the initial onset and spread of COVID-19, research suggests that the virus originated from bats [3]. COVID-19 is a febrile illness with a broad spectrum of respiratory involvement and may progress to involve multiple systems. Leukopenia, elevated c-reactive protein (CRP) levels, thrombocytopenia, and acute kidney injury were common laboratory findings associated with the disease [4-6].

Many drugs have been explored as potential treatments for COVID-19 [7], and research is ongoing for vaccine trials and therapies, including immunoglobulin, convalescent plasma, antiviral agents, and other modalities.

The novelty of the severe acute respiratory syndrome coronavirus-2 (SARS-CoV-2) steered the overwhelming trend of laboratory investigation during the follow-up of COVID-19 patients. Reviewing SARS-CoV-2 research data from time to time, we observed that too many investigations are performed to predict the outcome of patients, contributing to a substantial financial and psychological burden on these patients. Therefore, we evaluated the association between laboratory investigations and their effects on COVID-19 outcomes to determine if multiple laboratory evaluations are clinically justified.

## Materials and methods

We conducted an observational cohort study of COVID-19 patients treated as inpatients at the Shifa International Hospital (SIH) in Islamabad in April 2020. The ethical review board of SIH approved the study design. The study included inpatients of SIH diagnosed with COVID-19 via positive polymerase chain reaction testing in April 2020. The study excluded all surgical patients, pediatric patients (i.e., those younger than 15 years), chemotherapy patients, and patients with a hospital stay shorter than 48 hours. We also excluded any patients who did not receive follow-up laboratory evaluations.

We recorded study variables from patients' medical records. Laboratory values on admission and their trends during worsening of oxygen saturation were recorded and entered in IBM SPSS Statistics for Windows, Version 23.0 (IBM Corp., Armonk, NY, USA). Patient data were compared to United States standard values for interpretation. Severity was graded according to WHO criteria for COVID-19 severity. We calculated descriptive statistics for both qualitative and quantitative data. The effect of all variables on outcomes was calculated using chi-squared or Fisher's exact tests, and p-values <0.05 with 95% confidence interval were considered statistically significant.

## Results

A total of 51 patients with COVID-19 were enrolled. As shown in Table 1, most of our study patients were men older than age 50 with multiple comorbidities and resided in the Khyber Pakhtunkhwa district. A hospital stay of eight to 14 days was the most common, and most patients had severe disease. Only six patients (11.8%) died, however. Age, gender, comorbid conditions, and residence had no significant effect on disease severity, length of hospital stay, or mortality.

**Table 1:** Baseline characteristics of patients and frequencies of outcome (n = 51) KPK, Khyber Pakhtunkhwa; SD, standard deviation

Age (years)	Number of cases	%
<30	0	0
30 to 50	15	29.4
50 to 70	31	60.78
>70	5	9.8
Mean ± SD	57.2 ± 10.28	
Gender		
Male	41	80.4
Female	10	19.6
Residence		
KPK	19	37.3
Punjab	17	33.3
Islamabad	14	27.5
Baluchistan	1	2.0
Comorbidities		
Diabetes mellitus	9	17.6
Hypertension	7	13.7
Ischemic heart disease	1	2.0
Chronic kidney disease	1	2.0
Malignancy	2	3.9
Multiple	12	37.3
Nil	19	23.5
Severity		
Mild	12	23.5
Moderate	12	23.5
Severe	20	39.2
Critical	7	13.7
Mortality	6	11.8
Length of hospital stay		
1 to 7 days	19	37.3
8 to 14 days	20	39.2
15 to 28 days	12	23.5

During maximum symptoms, nine patients had blood pressure <110/70 mmHg and most of those patients (55.6%) had extended hospital stays (14 to 28 days; p = 0.055; Table 2). SpO_2_ value on admission strongly affected the length of hospital stay. Among patients presenting with <80% saturation, most of them had more extended hospital stays (14 to 28 days) than those with higher saturations (p = 0.000). We did not correlate oxygen saturation levels with severity of disease in our patients because a patient’s disease severity was categorized mainly on the basis of oxygen saturation, so increasing level of hypoxia was associated with more severe illness by default.

**Table 2:** Summary of effects of all variables on three outcomes BP, blood pressure; TLC, total leukocyte count; MCV, mean corpuscular volume; CRP, C-reactive protein; LDH, lactate dehydrogenase; ALT, alanine transaminase

Variable	Length of hospital stay p-values	Severity p-values	Mortality p-values
BP on admission	0.34	0.47	0.53
BP during maximum symptoms	0.055	0.28	0.55
Room air oxygen saturation on admission	0.000	NA	0.48
TLC on admission	0.27	0.15	0.15
TLC trend during worsening of symptoms	0.12	0.023	0.53
Platelets on admission	0.78	0.72	0.39
Platelets trend during worsening of symptoms	0.073	0.016	0.044
MCV on admission	0.96	0.58	0.43
MCV trend during worsening of symptoms	0.27	0.41	0.049
Hemoglobin on admission	0.73	0.05	0.75
Hematocrit on admission	0.87	0.13	0.56
Hematocrit trend during worsening of symptoms	0.062	0.54	0.51
CRP on admission	0.042	0.013	0.68
CRP during worsening of symptoms	0.62	0.169	0.067
D-dimer on admission	0.11	0.003	0.003
D-dimer during worsening symptoms	0.46	0.17	0.07
LDH on admission	0.50	0.23	0.015
LDH during worsening of symptoms	0.60	0.08	0.106
Ferritin on admission	0.13	0.317	0.28
Ferritin during worsening of symptoms	0.29	0.20	0.24
ALT on admission	0.17	0.029	0.015
ALT trend during worsening of symptoms	0.25	0.003	0.015

Platelet counts were most strongly associated with all study outcomes, followed by mean corpuscular volume (MCV), most associated with mortality, hematocrit with length of hospital stay, and hemoglobin and total leukocyte count (TLC) with severity. Platelet count levels were associated with worsening symptoms and more extended hospital stay (p = 0.07); reduced platelet count was associated with more severe illness and more mortality (p = 0.016, p = 0.044, respectively; Table 2).

MCV that increased during a worsening of symptoms affected the patient mortality (Table 2, Table 3) but not the severity and length of stay. Hemoglobin on admission affected the severity of illness: hemoglobin levels within reference range on admission were associated with more severe illness than low hemoglobin levels (p = 0.05; Table 2). Reduced hematocrit during a worsening of symptoms was associated with prolonged hospital stay with borderline significance (p = 0.063; Table 2).

**Table 3:** Association of variables affecting mortality (n = 51) MCV, mean corpuscular volume; CRP, C-reactive protein; ALT, alanine transaminase

Variable	Trend	Mortality		p-Value
		Died	Survived	
Platelets trend during worsening of symptoms	Increasing	1 (4.5%)	21 (95.5%)	0.044
	Decreasing	3 (37.5%)	5 (62.5%)	
	Static	2 (18.2%)	9 (81.8%)	
MCV trend during worsening of symptoms	Increasing	3 (50.0%)	3 (50.0%)	0.049
	Decreasing	0 (0.0%)	2 (4.4%)	
	Static	3 (9.1%)	30 (90.9%)	
CRP trend during worsening of symptoms	Increasing	3 (12.5%)	21 (87.5%)	0.067
	Decreasing	3 (42.9%)	4 (57.1%)	
	Static	0 (0%)	10 (100%)	
LDH on admission (units/L)	<200	0 (0%)	2 (100%)	0.015
	200-500	1 (2.9%)	33 (97.1%)	
	500-900	3 (27.3%)	8 (72.7%)	
	900-1,500	2 (50%)	2 (50%)	
D-dimers on admission (µg/mL)	200-500	0 (0%)	17 (100%)	0.003
	500-2,000	1 (0%)	10 (100%)	
	2,000-5,000	3 (8.3%)	11 (91.7%)	
	>5,000	2 (50%)	3 (50%)	
D-dimers trend during worsening of symptoms	Increasing	6 (23.1%)	20 (76.9%)	0.07
	Static	0 (0%)	15 (100%)	
ALT on admission (units/L)	<40	2 (4.9%)	39 (95.1%)	0.015
	40-80	1 (50%)	1 (50%)	
	>80	3 (37.5%)	5 (62.5%)	
ALT trend during worsening of symptoms	Increasing	3 (30%)	7 (70%)	0.015
	Decreasing	1 (100%)	0 (0%)	
	Static	2 (6.7%)	28 (93.3%)	

Lactate dehydrogenase (LDH) on admission affected mortality (p = 0.012), and most patients with LDH above 500 U/L died (Table 3). While LDH did not affect severity significantly, most patients with LDH ranging from 500 to 900 U/L had severe illness. C-reactive protein levels during a worsening of symptoms correlated to mortality with borderline significance: when CRP decreased during worsening symptoms, three of seven patients died (p = 0.067; Table 3).

D-dimer levels on admission above 2,000 ng/mL were associated with severe and critical illness in most cases (p = 0.003) and five of six mortalities (p = 0.003). During worsening of symptoms, all patients had either increasing or static D-dimer levels, and all six patients who died had increasing D-dimer levels (p = 0.07). Ferritin on admission and its trends did not affect any study outcomes.

Alanine transaminase (ALT) >80 mg/dL on admission was associated with mortality (p = 0.015) and with severe to critical illness (p = 0.029). More mortality (p = 0.015) and more severity (p = 0.003) were associated with ALT increasing during the worsening of symptoms. However, one patient had a fall in ALT during a worsening of hypoxia after an initial rise, and he died; this observation needs further examination from other studies.

## Discussion

Despite a rapidly increasing understanding of the pathogenesis of COVID-19, uncertainty remains about clinical symptoms and optimal management [8], so the need for further research is inevitable.

Lippi et al. observed that most published articles have discussed the clinical features and imaging findings of COVID-19 patients, yet few studies have addressed the diagnostic and prognostic value of abnormal laboratory findings [9]. Irrespective of its inherent definition [10], this branch of medical science is effectively involved in epidemiologic surveillance and prognosis determination.

We chose two clinical indices, five common complete blood count (CBC), and five non-CBC blood indices, and retrospectively followed them with the patient's clinical stage of severity and observed their impacts on outcomes. We observed that these indices fluctuated with the course of the illness - some had an impact on outcomes, and some did not. This observation can be used to rationalize laboratory testing.

Zhou et al. conducted a meta-analysis and concluded that comorbidities, including obesity, hypertension, and diabetes, were clinical risk factors for a severe or fatal outcome associated with COVID-19 [11]. Our study observations on age and gender align with the literature, but our findings for comorbidities and treatment did not align with the literature. Racial differences and lack of awareness might cause these observed differences.

For those who survived COVID-19, hypotension during maximum symptoms and oxygen saturation on admission <80% led to prolonged hospital stay, and hence correlated to poorer COVID-19 outcomes [12].

Yang et al. conducted a meta-analysis of nine studies and reported a low platelet count associated with an increased risk of severe disease and mortality [13]. Our findings align with Yang's, but we further conclude that reduced platelet count with worsening of the clinical situation was correlated with mortality, and increased platelet count correlated with more severe (non-critical) illness and more extended hospital stay. Therefore, platelet count trends in any direction might predict COVID-19 outcomes.

Most TLC studies reported that leukopenia or normal counts in these patients were associated with poorer outcomes [14]. Because most of our patients had elevated TLC (probably due to over-the-counter steroid use or superimposed infection), we observed more severe illness with rising TLC during a worsening of symptoms.

Lippi et al. concluded that in COVID-19 infection, a progressive decrease in the hemoglobin concentration might reflect a worse clinical progression [15]. Our study results showed that normal hemoglobin on admission, compared with low hemoglobin, was associated with more severe disease. This finding may be more linked to the generally high financial group coming to private hospitals and the high-altitude residence of most study subjects, so relative anemia cannot be excluded. Additional evaluation with meta-analysis from different financial and altitude groups is warranted.

MCV rise with disease progression might be indicative of the development of leukoerythroblastic picture with disease course and, therefore, mortality in our study as Mitra et al. described in their article. However, they were only reporting the findings of a single patient with COVID-19, and they aimed to describe the finding of leukoerythroblastosis in this viral infection [16].

In our study, hospital stay was prolonged with decreasing hematocrit with worsening symptoms, just as Wang et al. concluded that low hematocrit was a predictor of poor outcomes [17].

Sun et al. noted in COVID-19 patients that liver function tests, including aspartate transaminase, ALT, gamma-glutamyl transferase, and LDH, were elevated [18]. Huang associated elevated ALT with more severe illness [4]. Our study results endorse these findings and suggest that ALT is a robust prognostic marker.

Ye et al. concluded that D-dimer's initial and peak values in deceased COVID-19 patients were higher [19]. We found D-dimer as one of the more critical prognostic factors affecting our study outcomes.

Dong et al. reported that LDH levels in COVID-19 patients who died were significantly higher than those who survived [20]. Our study findings endorsed Dong's findings, as we found LDH on admission and its trend as a useful prognostic marker.

While Cheng et al. suggested that ferritin was associated with poor prognosis in COVID‐19 patients [21], Feld et al. reported that elevated ferritin levels were not accurate predictors of outcomes [22]. Our study results align with Feld's finding, indicating against testing ferritin levels repeatedly as a prognostic marker of COVID-19 disease.

Chen et al. demonstrated that the plasma CRP level was positively correlated to the severity of COVID-19 [23]. In our study, elevated CRP on admission was associated with more severe disease and extended hospital stay. During worsening of symptoms, a reduction in CRP was significantly correlated with more mortality, likely indicating a decline in the patient's immune response.

Our small sample size limited our study, and we recommend larger trials to confirm our findings. We did not correlate our findings with disease complications due to missing data, so future studies should compensate for this accordingly.

## Conclusions

We evaluated the association between laboratory investigations and their effects on COVID-19 outcomes to determine if multiple laboratory evaluations are clinically justified. Patient age, gender, comorbid conditions, drugs, residence, and ferritin levels did not affect disease outcomes. Hemoglobin, platelet count, MCV, CRP, D-dimer, ALT, LDH, hypoxia, and hypotension were all correlated to disease outcomes. Therefore, these factors are sufficient, justifiable laboratory examinations for COVID-19 patients, especially in poor countries where resources and funds for laboratory examinations are limited.
